# Microbial Danger Signals Control Transcriptional Induction of Distinct MHC Class I L Lineage Genes in Atlantic Salmon

**DOI:** 10.3389/fimmu.2019.02425

**Published:** 2019-10-11

**Authors:** Steingrim Svenning, Agata T. Gondek-Wyrozemska, Yorick Andreas van der Wal, Børre Robertsen, Ingvill Jensen, Jorunn B. Jørgensen, Eva-Stina Edholm

**Affiliations:** ^1^Norwegian College of Fishery Science, Faculty of Biosciences, Fisheries & Economics, University of Tromsø—The Arctic University of Norway, Tromsø, Norway; ^2^Vaxxinova Research & Development, Vaxxinova GmbH, Münster, Germany

**Keywords:** Atlantic salmon, MHC class I, L-lineage, SAV3, *Piscirickettsia salmonis*

## Abstract

Antigen processing and presentation by major histocompatibility complex (MHC) molecules is a cornerstone in vertebrate immunity. Like mammals, teleosts possess both classical MHC class I and multiple families of divergent MHC class I genes. However, while certain mammalian MHC class I-like molecules have proven to be integral in immune regulation against a broad array of pathogens, the biological relevance of the different MHC class I lineages in fish remains elusive. This work focuses on MHC class I L lineage genes and reveals unique regulatory patterns of six genes (*Sasa*-*lia, Sasa*-*lda, Sasa*-*lca, Sasa*-*lga, Sasa*-*lha*, and *Sasa*-*lfa*) in antimicrobial immunity of Atlantic salmon (*Salmo salar L*.). Using two separate *in vivo* challenge models with different kinetics and immune pathologies combined with *in vitro* stimulation using viral and bacterial TLR ligands, we show that *de novo* synthesis of different L lineage genes is distinctly regulated in response to various microbial stimuli. Prior to the onset of classical MHC class I gene expression, *lia* was rapidly and systemically induced *in vivo* by the single-stranded (ss) RNA virus salmonid alpha virus 3 (SAV3) but not in response to the intracellular bacterium *Piscirickettsia salmonis*. In contrast, *lga* expression was upregulated in response to both viral and bacterial stimuli. A role for distinct MHC class I L-lineage genes in anti-microbial immunity in salmon was further substantiated by a marked upregulation of *lia* and *lga* gene expression in response to type I IFNa stimulation *in vitro*. Comparably, *lha* showed no transcriptional induction in response to IFNa stimulation but was strongly induced in response to a variety of viral and bacterial TLR ligands. In sharp contrast, *lda* showed no response to viral or bacterial challenge. Similarly, induction of *lca*, which is predominantly expressed in primary and secondary lymphoid tissues, was marginal with the exception of a strong and transient upregulation in pancreas following SAV3 challenge Together, these findings suggest that certain Atlantic salmon MHC class I L lineage genes play important and divergent roles in early anti-microbial response and that their regulation, in response to different activation signals, represents a system for selectively promoting the expression of distinct non-classical MHC class I genes in response to different types of immune challenges.

## Introduction

Major histocompatibility complex (MHC) class I genes are present in all classes of jawed vertebrates and encode a diverse family of molecules that play integral roles in the host‘s immune defense. Included in the MHC class I family are highly polymorphic classical MHC class I molecules that primarily present intracellularly derived peptides to conventional CD8^+^ αβ T cells. Ancillary to classical MHC class I genes, jawed vertebrates also possess variable numbers of non-polymorphic MHC genes, that in humans have been termed “non-classical MHC” for those genes located within the MHC loci and “MHC class I-like” for those located outside the MHC proper. Although structurally similar to classical MHC class I, many non-classical/MHC class I-like molecules have functions other than peptide presentation, ranging from host homeostasis to immune regulation [reviewed in (1)]. In general, non-classical/ MHC class I-like molecules are largely considered part of the innate immune responses, particularly as receptor ligands for natural killer (NK) cells (2, 3), γ/δ T cells and semi-invariant T cells (4–6).

To date, six different MHC class I lineages have been described in bony fish; U, Z, S, L, P [reviewed in (7)] and most recently, H (8). In the absence of functional data, teleost MHC class I lineages have mainly been distinguished based on allelic polymorphism, phylogeny, and conservation or loss of the eight canonical peptide-anchoring residues (9, 10). Teleost classical MHC class I genes all belong to the U lineage. In addition, U lineage genes with more limited polymorphism and a more restricted expression pattern have been described (11, 12). While the U and Z lineage genes have, in most cases, maintained the hallmark MHC class I peptide anchoring residues in their α1-α2 domains, the S, L, and P lineage genes lack these residues, suggesting that these molecules bind non-peptide ligands or no ligands (13). Of note, L lineage α1-α2 domains possess the highest hydrophobicity of all five teleost MHC class I families described to date, which has prompted speculations that this lineage might be involved in presentation of glycolipids or other hydrophobic ligands in a manner similar to mammalian CD1 molecules (13), and reviewed in (7). Although distinct and divergent MHC class I gene families have been described in a number of teleost species, very little is known about their biological relevance and potential roles in antigen presentation and regulation of T and NK cells.

In Atlantic (Atl.) salmon, in addition to a single classical MHC class I gene [*Sasa*-*uba*, (14)], located on chromosome 27, *in silico* analysis have uncovered eighteen functionally expressed MHC class I loci scattered throughout the genome (13). These include six L lineage genes; *Sasa*-*lda, Sasa*-*lia, Sasa*-*lca, Sasa*-*lfa, Sasa*-*lga*, and *Sasa*-*lha* that are distributed across chromosomes 11, 12, 21, and 26 (7). Among these, *lda, lia*, and *lca* are located on separate chromosomes, while *lfa-lga-lha* are closely arranged in tandem on chromosome 26. In general while the structure of L lineage genes resembles classical MHC class I, all six L lineage genes have a unique intron-exon structure with a 2 exon gene organization: the first exon encode the leader signal peptide and the second the combined α-chain, transmembrane domain and cytoplasmic tail (15). L lineage genes share between 58–82% amino acid identities, with *lia* representing the most divergent isoform. The vast majority of sequence disparity is concentrated in the α1-α2 domains, while the α3 domains are highly conserved among the six genes. In terms of gene expression, L lineage transcripts, as estimated by transcriptome analysis of an adult Atl. salmon, have been detected in most tissues, albeit at levels significantly lower compared to the classical MHC class I gene, *uba* supporting their classification as non-classical MHC class I (13).

In these studies, we considered the possibility that microbial pathogens might modulate expression of MHC class I L lineage genes. Further, while these genes are expressed at relatively low levels in healthy fish, we hypothesize that individual L lineage genes are induced in response to specific immune challenges, thereby contributing to Atl. salmon immune surveillance and response. We initially focused on two ecologically relevant salmonid pathogens with different pathologies and infection kinetics. This included salmonid alphavirus 3 (SAV3), a membrane enveloped, single-stranded (ss) RNA virus causing pancreatic disease in salmonids [reviewed in (16)] and *Piscirickettsia salmonis*, an intracellular gram negative bacterial pathogen capable of infecting a variety of salmonid species, including Atl. salmon, resulting in the development of piscirickettsiosis [reviewed in (17)]. To investigate the potential roles for various L lineage genes during a microbial infection, individual transcriptional profiles were compared to those of the classical MHC class I gene. In addition, transcriptional profiles of type I interferons (IFNs) were included to evaluate the immune regulatory context of the two challenge models. We found that SAV3 as well as dsRNA and ssRNA viral mimics, strongly induced expression of *lia, lga* and to a lesser extent *lha*, but not *lda, lca*, or *lfa*. Comparably *Piscirickettsia salmonis* infection predominantly resulted in upregulation of *lga*. Further, we found that recombinant type I IFNa strongly upregulated *lia* and *lga* but failed to upregulate *de novo* synthesis of *lha*. These studies define a potential immunological function of distinct L lineage genes in immune surveillance, revealing that transcriptional induction of these genes is differentially modulated in response to viral and bacterial danger signals.

## Materials and Methods

### Experimental Animals and Infection Protocols

For basal tissue expression and leucocyte isolation, healthy 60–70 gram pre-smolt Atlantic salmon (*Salmo salar* L.) from Aqua Gen (Aqua Gen, Kyrksæterøra, Norway) were obtained from the Tromsø Aquaculture Research Station (Tromsø, Norway). Fish were kept at 10°C in tanks supplied with running filtered water, natural light, and fed on commercial dry feeds (Skretting, Stavanger, Norway). Challenge experiments were performed at the Tromsø Aquaculture Research Station and all animals were treated in accordance with relevant guidelines and regulations given by the Norwegian Animal Research Authority. The experimental protocols used for live fish experiment were based on the Animal Welfare Act (https://www.regjeringen.no/en/dokumenter/animal-welfare-act/id571188/). All experiments were approved by the national committee for animal experimentation (Forsøksdyrutvalget, Norway).

#### Salmon Alpha Virus 3 (SAV3) Challenge

Salmonid alphavirus subtype 3 (SAV3) (PDV-H10-P3A) was obtained from Øystein Evensen (Faculty of Veterinary Medicine and Biosciences, Norwegian University of Life Sciences, Norway). The virus was propagated in CHH-1 cells, derived from heart tissue of a juvenile chum salmon (*Onchorhynchus keta)*, in L-15 medium with 100 U/mL penicillin, 100 μg/mL streptomycin, and 5% FBS at 15°C. Virus titer was determined by the TCID_50_ method. For the SAV3 challenge experiment, pre-smolt (60–70 gram) that had been maintained in 10°C fresh water with a 24 h of light and fed to satiation for 7 weeks prior to challenge were used. Fish were randomly allocated to two tanks and one group received intra peritoneal (i.p) injection with 100 μl SAV3 (1 × 10^5^ TCID_50_) and the second group was i.p injected with an equivalent volume of PBS. Tissue samples were collected at 3, 8, and 14 days post infection.

#### *Piscirickettsia salmonis (P. salmonis)* Challenge

The *Piscirickettsia salmonis (P. salmonis)* EM-90-like strain PM15972 was obtained from ADL Diagnostics Chile. *P. salmonis* was cultivated on PSA agar plates [as described in (18)] and harvested after 5 days. The harvested bacterial suspension was adjusted to an OD_600_ of 0.890 (1 × 10^8^ CFU/ml) and diluted to the challenge dose of 3 × 10^6^ CFU/fish. For the *P. salmonis* challenge experiment, pre-smolt (50 gram) that had been maintained in 10°C fresh water with 24 h of light and fed to satiation for 7 weeks prior to challenge were used. Fish were randomly allocated to two tanks, one group received an i.p. injection with 100 μl *P. salmonis* (3 × 10^6^ CFU) while the second group was i.p injected with an equivalent volume of PBS. Tissue amples were collected at 2, 7, and 14 days post infection. Fish were kept in fresh water at 10°C for the duration of the experiment.

### Primary Cell Isolation

Leukocytes from Head-kidney (HK), spleen, and peripheral blood (PB) were isolated on Percoll (GE Healthcare) gradients. Briefly, HK and spleen were sampled aseptically and kept on ice-cold transport medium (L-15 medium with 10 U/ml penicillin, 10 μg/ml streptomycin, 2% fetal bovine serum (FBS), 20 U/ml heparin) until homogenization on 100-μm cells strainers (Falcon). The resulting cell suspensions were layered on 25/54% discontinuous Percoll gradients and centrifuged at 400 × *g* for 40 min at 4°C. Cells at the interface were collected and washed twice in L-15 medium. PB was collected from the caudal vein using heparinized vacutainer tubes and diluted immediately two fold in ice-cold transport medium. The resulting suspension was layered on a 54% Percoll gradient, centrifuged, and harvested as above. Cells were counted using an automatic cell counter (Countess II Automated cell counter, Thermo Fisher, cat. nr. AMQAF1000).

### Cell Lines

Atlantic salmon kidney (ASK) cells (ATCC-CRL-2747; American Type Culture Collection) were grown at 20°C in L.-15 medium containing 1XMEM Nonessential Amino Acid Solution (Invitrogen), 100 U/ml penicillin, 100 μg/ml streptomycin, and 15% FBS.

### *In vitro* Cell Stimulations and Reagents

Leucocytes were isolated as described above, diluted to a concentration of 2 × 10^6^ cells /mL and seeded in IL-15^+^ (L-15 supplemented with 5% FBS and penicillin/streptomycin) in 24 well plates (Nunclon Delta Surface, Thermo Scientific), stimulated with either 10 μg/ml HW+LMW Poly I:C (Invivogen, tlrl-pic and tlrl-picw) at a 1:1 ratio; 2 μg/ml R848 (Invivogen, tlrl-r848); or varying concentrations; 0.5, 1, 2, and 4 μM CpG ODNs [Invivogen (ODN 2216 (A), ODN 2243 (A control), ODN 2006 (B), ODN 2006 (B control)]; or left untreated (medium alone) and cultivated at 14°C for 12, 24, and 48 h.

### Stimulation of Primary HK Leucocytes and ASK Cells With Recombinant IFNa

Recombinant interferon was available from previous work (19, 20). Briefly, a plasmid encoding the open reading frame of Atlantic salmon IFNa1, pCR3.1SasaIFN-α1 (20) was used and sub confluent HEK293 cells were transfected with 500 ng IFNa plasmid using 7 μl FugenHD Transfection reagent (Roche Diagnostics). Cell media containing rIFNa was harvested 48 h post transfection, centrifuged for 5 min at 3000 × g, filtered through a 0.45-μm filter, and frozen in aliquots at −70°C until use. For stimulation 2 × 10^6^ head kidney leucocytes (HKL) were seeded in IL-15^+^ (L-15 supplemented with 5% FBS and penicillin/streptomycin) in 24 well plates (Nunclon Delta Surface, Thermo Scientific), stimulated with 500 U of rIFNa or media alone and cultivated at 16°C for 12, 24, and 48 h prior to harvesting. ASK cells were seeded at 500,000 cells per well in 6-well plates in L.-15 medium containing 1 X MEM Nonessential Amino Acid Solution (Invitrogen), 100 U/ml penicillin, 100 μg/ml streptomycin and 15 % FBS and stimulated with either 200 or 500 U rIFNa or left untreated.

### Quantitative PCR and Transcript Analysis

Total RNA from tissues, primary leucocytes, and stimulated leucocytes was isolated using the RNeasy Mini Kit (Qiagen) following the manufacturer's recommendation. On-column DNase digestion was performed using RNase-free DNase set (Qiagen), and RNA was quantified using NanoDrop (ND 1000 Spectrophotometer). One microgram total RNA was then further treated using DNase I to remove all residual genomic DNA. Twenty microliter cDNA reactions with 350 ng total RNA were synthesized using TaqMan reverse transcription reagents (Applied Biosystems) using random hexamer primers under the following conditions: 25°C for 10 min, 37°C for 30 min and 95°C for 5 min. cDNA samples were diluted 1:2 and stored at −20°C until use. Quantitative PCR (qPCR) was run as 10 μl duplicate reactions on a 7500 Fast Real-Time PCR Systems (Applied Biosystems) according to standard protocol. All primers were validated and primer sequences are supplied in sTable 1. To ensure that the individual L lineage primers were specific the resulting qPCR product was cloned and sequenced. For each primer pair and tissue/cell a negative control (no template) and a no reverse transcriptase control RT (–) was performed. A threshold difference of at least six quantification cycles (*C*_q_) between RT (+) and RT (–) was used as a cut-off. Ct values >37 were rejected. Parameters were as follows: 2 min at 95°C followed by 40 cycles of 95°C for 15 s and 60°C for 1 min. A melt curve analysis was also performed to ensure that a single product had been amplified. Relative quantitative PCR gene expression analysis was performed using the ΔΔCt method. Expression of the different genes was examined relative to the endogenous EF1-αB control and normalized against the lowest observed tissue expression for each gene. Relative expression (zero-hour samples) was calculated using the 2^−ΔCq^ method where ΔCq was calculated by subtracting the EF1aB Cq value from the target gene Cq value. For infected tissues and stimulated cells, fold change was calculated using the non-treated fish and cells from each tissue and time point as a control.

### Statistical Analysis

All quantitative data were based on duplicate measurements from a minimum of *four fish n* ∈ [4;8] and were analyzed in GraphPad Prism 8. Statistical evaluation were performed using Tukey‘s multiple comparisons test following a significant one-way ANOVA. Correlation among MHC class I L lineage expression, pathogen load and interferon expression was determined using the Pearson Correlation coefficiency (*p* = 0.05) calculated from the relative expression of each gene normalized to EF1-αB. For all analysis a *p* value > 0.05 was considered significant.

### Sequence Analysis

All L lineage sequences and their putative promoter regions were extracted from the SalmonBase (https://salmobase.org/). Specific promoter region elements were identified via MatInspector (0.85 matrix/identity cutoff) and manual analysis. For transcriptome analysis sequence read analysis deposited in the NCBI Bioproject database were analyzed using the BLASTn SUIT-SRA interface and Reads Per Kilobase Per Million (RPKM) were calculated based on hits that mapped with high stringency (i.e., greater than 98% identity) and fold change were calculated compared to respective control datasets when appropriate. The transcriptome (SRA) accession numbers used are as follows: PRJNA472087 for infectious salmon anemia virus (ISAV) challenge (21), and PRJNA543940 for SAV3 challenge.

## Results

### Tissue Specific Expression Patterns of MHC Class I L Lineage Genes

As a first step to understand the biological roles of MHC class I genes in teleosts basal transcript levels of MHC class I L lineage genes were determined in lymphoid and non-lymphoid tissues from pre-smolt Atlantic salmon using qRT-PCR (sFigure 1). Congruent with previous reports based on transcriptome analysis of a single adult Atl. salmon (13) modest constitutive expression of *lia, lda, lca, lga*, and *lha* was evident in primary and secondary lymphoid organs (thymus, spleen, and head-kidney), as well as the gill and gut associated tissues. Further, and again consistent with previous reported transcriptome analysis the overall relative expression of all six L lineage genes was markedly lower compared to classical MHC class I (*uba*). On average Ct values for *uba* in the gill was 18.4 (SE ± 0.1) compared to 32.2 (SE ± 0.3), 28.7 (SE ± 0.3), 34 (SE ± 0.4), 27.5 (SE ± 0.2), 28.6 (SE ± 0.4), and 26.2 (SE ± 0.3), for *lia, lda, lca, lga, lha*, and *lfa* respectively. Among the L lineage genes, and representative across all tissues *lga*, and *lha*, had the highest basal expression whereas baseline expression of *lia* and *lca* were significantly lower; 17, 28, and 430-fold lower for *lia* and 20, 7, and 1,136-fold lower for *lca* compared to *lga* in thymus (*P* < 0.001), head kidney (HK, *P* <0.001), and gill (*P* < 0.001) respectively. In terms of tissue distribution, the qPCR based expression patterns mirrored that of previous reported transcriptome data (13). Briefly, basal expression of *lga* and *lia* to some extent resembled that of *uba*, with relatively high expression observed in gills and gut. Similarly, *lda* was relatively highly expressed in gill, however, transcript levels of *lda* were also pre-dominant in kidney and liver. For *lha*, ubiquitous expression was observed in most tissues examined; although, transcription of this gene was notably lower in skin, heart and liver. In sharp contrast, *lca* displayed a more restricted expression pattern and transcript levels were significantly higher in spleen and head kidney compared to all other tissues examined. The most restricted expression pattern was observed for *lfa*. *Lfa* transcripts were only consistently detected above threshold levels in the gill, skin, dorsal fins, and thymus. Low levels of *lfa* transcripts were also detected in the gut and liver, but did not reach threshold levels (Ct < 37) in all fish.

### Potential Regulatory Elements in the 5′-End Flanking Regions of L Lineage Genes

**Figure 1:**
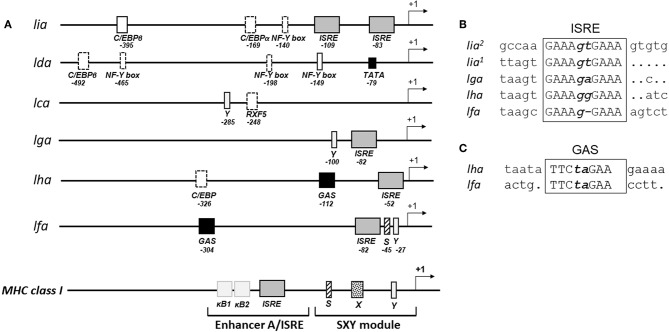
Distinct combinations of response elements in the promoter regions of different L lineage genes. **(A)** Schematic representation of the putative promoter regions for MHC class I L lineage genes. Five hundred nucleotides upstream of each L lineage gene were identified using SalmonBase and potential regulatory elements were identified via MatInspector (0.75 matrix/identity cutoff) and manual analysis. Regulatory elements are indicated as boxes, with solid lines representing complete conservation with the respective consensus sequences and dotted lines representing a partially conserved promoter element. Arrows indicate transcriptional initiation sites, interferon stimulated response elements (ISRE) are shown as gray boxes and gamma interferon activation sites (GAS) are shown as black boxes. A model illustrating the prototypical MHC class I promoter elements common to many vertebrate species is shown at the bottom. **(B)** Alignment of the different MHC class I L lineage ISRE motifs [consensus GAAA(N_1−2_)GAAA] and **(C)** GAS motifs [consensus TTC(N_2−4_)GAA].

Constitutive and inducible transcription of classical MHC class I genes is tightly regulated by a variety of transcription factors that interact with conserved cis acting regulatory promoter elements. These include the MHC specific SXY-module, interferon stimulated response elements (ISRE) and Enhancer A (κB1 and κB2). A relatively high conservation of these key regulatory elements has been identified among putative proximal promoter regions of classical MHC class I in rainbow trout (22, 23), Atlantic salmon (24) grass carp (25), and channel catfish (26) including a full or partial conservation of the SXY module and the presence of at least one ISRE. To gain insight into the transcriptional regulation of MHC class I L lineage genes, we analyzed the proximal promoter region 500 nucleotides upstream of the translation start site of the different L lineage genes. A combination of manual examination and annotation was performed with reference to SXY modules, ISRE [consensus sequence GAAA-(N_1−2_)-GAAA] and gamma interferon activation site [GAS; consensus sequences TTC-(N_2−4_)-GAA]. As expected, no conserved SXY motifs were found in the promoter region of any of the MHC class I L lineage genes. However, ISRE elements, which control transcription of genes induced by type I IFNs, were identified in *lia, lga, lha*, and *lfa* but not in *lda* or *lca* sequences (Figure 1 and sFigure 2). Notably, the *lia* promoter contained two proximally located identical ISRE elements. Comparably, in *lha* and *lfa* a GAS element, which is the main promoter elements for IFNγ induction, was identified 45 and 214 nucleotides upstream of the respective ISRE sequence. In addition, conserved CCAAT binding boxes were identified in *lia* and *lda* promoter regions along with binding sites for the CCAAT/Enhancer Binding Protein β (C/EBPβ) transcription factor. Taken together these data suggest that type I and type II IFNs have distinct roles in regulating the gene expression of different L lineage genes, implying that these genes are induced in response to a microbial challenge.

### Viral and Bacterial Immune Challenges Elicit Distinct MHC Class I L Lineage Gene Expression Patterns *in vivo*

**Figure 2:**
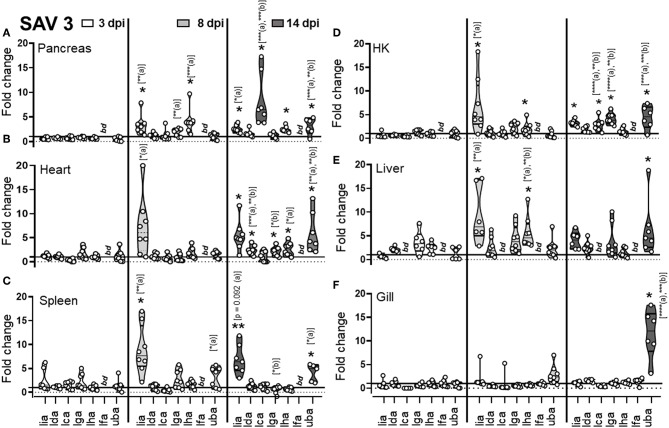
Expression of MHC class I L lineage genes in different tissues of Atlantic salmon following i.p SAV3 challenge. Pre-smolt Atlantic salmon were intra peritoneal (i.p) injected with 100 μl SAV3 (1 × 10^5^ TCID_50_) or PBS and samples were collected at 3 (white), 8 (light gray), and 14 days (dark gray) post infection. **(A–F)** Fold change in the expression of L lineage MHC class I genes (*lia, lda, lca, lga, lha*, and *lfa*) and classical MHC class I (*uba*) in different tissues at various times post challenge (*n* ϵ 8), relative to the control groups from the same time point (*n* ϵ 4). Each dot represents an individual fish, and the line intersecting the y-axis at 1 represents the unstimulated control that the fold change of the treatments is in relation to. Asterisks above each violin plot indicates the strength of significance compared to PBS injected controls, and letters above each violin represent statistical differences among the different time points for each individual gene (*a* = 3 dpi; *b* = 8 dpi, and *c* = 14 dpi), *p < 0.05, **p < 0.01, ***p = 0.0001, ****p < 0.0001, *bd*, below detection.

**Figure 3:**
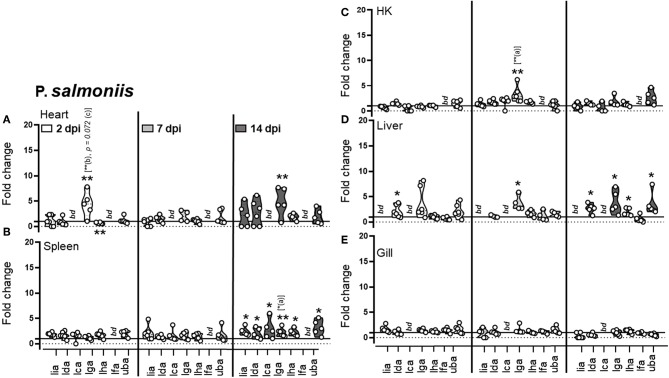
Expression of MHC class I L lineage genes in different tissues of Atlantic salmon following i.p. *P. salmonis* challenge. Pre-smolt Atlantic salmon were intra peritoneal (i.p) injected with 3 × 10^6^ CFU *P. salmonis* or an equivalent volume of PBS and samples were collected at 2, 7, and 14 days post infection. **(A–E)** Fold change in the expression of different L lineage MHC class I genes (*lia, lda, lca, lga, lha*, and *lfa*) and classical MHC class I (*uba*) in different tissues at various times post challenge (*n* ϵ 6) relative to the control groups from the same time point (*n* ϵ 6) is shown. Each dot represents an individual fish and the line intersecting the y-axis at 1 represents the unstimulated control that the fold change of the treatments is in relation to. Asterisks above each violin plot indicates the strength of significance compared to PBS injected controls, and letters above each violin represent statistical differences among the different time points for each individual gene (*a* = 2 dpi; *b* = 7 dpi, and *c* = 14 dpi), *p < 0.05 and **p < 0.01. bd, below detection.

To investigate the relevance of MHC class I L lineage genes during immune responses, we used two different salmonid pathogens: the alphavirus SAV3 and the intracellular bacterium *P. salmonis*. Given that nonclassical/MHC class I-like genes in mammals often have specialized functions in the innate immune responses, we anticipated that infection with either SAV3 or *P. salmonis* would result in transcriptional modulation of L-lineage genes during the early stages of infection. Accordingly, we intraperitoneally (i.p.) infected pre-smolt Atl. salmon with SAV3 or *P. salmonis*. Tissues were collected at: 3, 8, and 14 days post infection (dpi) for SAV3 and at 2, 7, and 14 dpi for *P. salmonis* challenged fish. Tissues from mock infected fish (PBS) were collected at matching time points and used as controls. To evaluate pathogen loads and dissemination, the relative expression of SAV3 and *P. salmonis* transcripts were determined using qPCR with primers specific to the nonstructural protein 1 (nsP1) for SAV3 (27) and 16S rRNA for *P. salmonis* (28) (sFigures 3, 4). Transcript levels of L lineage genes were quantified in relevant organs using qPCR and contrasted with that of classical MHC class I (Figures 2, 3).

For SAV3, as expected and congruent with the pancreas and heart being the main sites of SAV3 replication, viral specific transcripts were readily detected in these tissues and for heart increased significantly (*P* = 0.006) from 8 to 14 dpi (sFigure 3). In addition, viral transcripts in the spleen and gill were initially detected at 8 dpi and increased significantly by 14 dpi (*P* = 0.0059 and *P* = 0.037, respectively). In HK and liver SAV3 transcripts were detected by 8 dpi albeit at relatively low levels (sFigure 3). In regards to MHC class I L lineage genes, the most dramatic upregulation, following SAV3 challenge, was observed for *lia*. In the main target organs for SAV3 (pancreas and heart), the central lymphoid organs (spleen and head-kidney), and the liver *lia* expression was barely detectable in uninfected animals, became significantly elevated at 8 dpi, and was maintained from 8 to 14 dpi, indicating a systemic upregulation of *lia* in response to i.p. SAV3 infection (Figures 2A–E). In addition, *lga* and *lha* transcript levels were elevated in response to SAV3 challenge in a tissue specific manner. Both *lga* and *lha* transcripts increased compared to that of control fish in head kidney but with different kinetics; *lha* RNA levels peaked at 8 dpi and then returned to near baseline levels by 14 dpi, while *lga* expression was elevated at 8 dpi and reached significance by 14 dpi (Figure 2). In the heart, *lha* and *lga* levels were modestly upregulated compared to control fish by 14 dpi and in the pancreas *lha* levels were significantly elevated at 8 dpi and maintained by 14 dpi, no increase in *lga* expression was observed in the pancreas. *Lha* was also transiently upregulated in liver, peaking at 8 dpi and then returning to baseline. For *lca* and *lda*, with the exception of a marked upregulation of *lca* in pancreas at 14 dpi, transcriptional induction in response to SAV3 was modest. *Lfa* expression was undetectable (Ct cut-off set to 37) in all tissues except gill where no upregulation was observed. In fact, despite detectable viral transcripts in the gill, no increase in any L lineage gene transcript were observed in this tissue (Figure 2F). Consistent with previous reports (29, 30) classical MHC class I transcripts were elevated in all tissues (including gill) in response to SAV3 infection. However, significant upregulation was not observed until 14 dpi, indicating that *lia* and *lha de novo* synthesis occurs prior to that of classical MHC class I. To obtain additional evidence regarding the roles of specific L lineage genes in anti-SAV immunity, we examined whether SAV3 RNA levels correlated with induced L lineage mRNA. Positive correlation, as measured by the Pearson correlation coefficient (*p* > 0.05), was consistently found between *lia* and viral transcripts in the relevant tissues (sTable 2). A positive correlation between *lga, lha*, and viral RNA was also apparent in the main target organs (pancreas and heart) as well as the liver. Comparably, strikingly different L lineage expression patterns emerged in response to *P. salmonis* infection. Consistent with a systemic infection, *P. salmonis* transcripts were detected in both lymphoid and non-lymphoid tissues as early as 3 dpi with the highest bacterial burden observed in spleen and heart (sFigure 4). In general, *P. salmonis* infection resulted in none or marginal induction of L lineage genes with one notable exception, *lga* transcript levels were upregulated in all tissues examined except gill (Figures 3A–E). Compared to uninfected control fish, the highest upregulation was in the heart, where *lga* transcript levels increased > 4-fold at 2 dpi followed by a transient decline in expression at 7 dpi and return to elevated levels by 14 dpi. Similarly, HK *lga* expression was transiently elevated at 7 dpi while in the liver *lga* levels were maintained at around 3-fold higher compared to controls at 7 and 14 dpi. In the liver we also observed bimodal induction pattern of *lda* gene expression with an initial elevation at 2 dpi followed by a decline in transcript levels to near baseline at 7 dpi and an upregulation at 14 dpi.

To substantiate these observations and to gain preliminary insight into the kinetics of L lineage gene expression and potential involvement in other viral diseases we extended our expression analysis by searching currently available bioprojects deposited into the NCBI database. *In silico* analysis of 35 sequence read analysis (SRA) sets (bioproject PRJNA543940) from heart of pre-smolts infected with SAV3 (sText 1) revealed a slight, albeit not statistically significant, upregulation at the 4 week time point that decreased to near baseline by 10 wpi (sTable 3). While these observations need to be further substantiated it may indicate that transcriptional induction of L lineage genes predominantly reflects involvement during the early immune response. In support of this, analysis of twelve SRA sets from Atl. salmon infected with the orthomyxoviridae Infectious salmon anemia virus (ISAV) [Accession number: PRJNA472087 and (21)] revealed that, similar to SAV3 infection, *lia* transcript levels were elevated as early as 3 days post confirmed ISAV outbreak in HK and liver but not in gill (sText 1B, sTable 4) Collectively, these observations indicate that *lia* in particular but also, *lga* and *lha* may be involved in the early antiviral immune response against SAV3, while *lga* may have a role in anti-bacterial immune responses against intracellular bacteria. Taken together these data suggest that different L lineage genes are distinctly, both temporally and spatially, regulated in response to microbial infections, suggesting divergent functional roles.

### The Interplay Between MHC Class I L Lineage Transcriptional Induction and the Type I Interferon Response

A common way to enhance classical MHC class I expression and presentation of intracellular antigens is via immunomodulatory actions mediated through the type I IFN system. Given the presence of multiple interferon response elements in the promoter regions of *lia, lga*, and *lha* and the upregulation of these genes following SAV3 and/or *P. salmonis* infection, we next investigated whether there was a correlation among type I interferon expression and L lineage gene induction. Type I IFNs in Atl. salmon are represented by a large repertoire of IFN genes encompassing at least 6 different classes (IFNa, IFNb, IFNc, IFNd, IFNe, and IFNf) with several subtypes for each (31). Several studies have shown up-regulation of IFNa by virus infection in live fish (32–34), and reviewed in (31) while the *in vivo* effects of the other IFN classes remains less well understood. In this study we focus on IFNa, IFNc, and IFNb which have demonstrated antiviral activities (19, 35) and IFNd which, to date, has no clear role in antiviral defense in Atl. salmon. Primer sequences specific for IFNa1 and IFNa2, for all IFNb and all IFNc genes were used (19), revealing two very different type I interferon induction patterns in the different challenge models.

**Figure 4:**
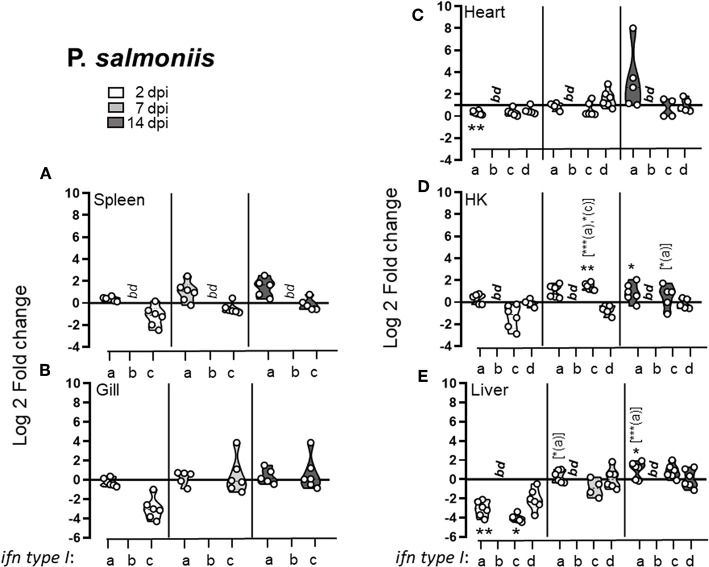
*Piscirickettsia salmonis* infection leads to early downregulation of the type I IFN response. Pre-smolt Atlantic salmon were intra peritoneal (i.p) injected with 3 × 10^6^ CFU *P. salmonis* or an equivalent volume of PBS and samples were collected at 2, 7, and 14 days post infection. Log 2 fold change of type I IFNa; IFNb, IFNc, and IFNd in different tissues; **(A)** spleen, **(B)** gill, **(C)** heart, **(D)** head kidney (HK), and **(E)** liver at different times post challenge [*n* ϵ (5;6)] relative to the control groups from the same time point [*n* ϵ (5;6)] is shown. Each dot represents an individual fish and the line intersecting the y-axis at 0 represents the unstimulated control that the fold change of the treatments is in relation to. Asterisks above each violin plot indicates the strength of significance compared to PBS injected controls, and letters above each violin represent statistical differences among the different time points for each individual gene (*a* = 3 dpi; *b* = 7 dpi, and *c* = 14 dpi), *bd*, below detection, *p < 0.05, **p < 0.01, ***p = 0.0001. Primer sequences that are conserved for IFNa1 and IFNa2, for all IFNb and all IFNc subtypes were used (19).

In general *P. salmonis* infection failed to provoke a type I IFN response and a very modest (at the most 2.5-fold) induction of type I IFNs were observed during the later stage of infection (Figure 4). Indeed, during the early stages of infection, a general downregulation of type I IFNs was observed. At 3 dpi, IFNc transcript levels were lower in infected fish compared to controls in all tissues examined, with significant down-regulation in the liver, where a concomitant down-regulation of IFNa was observed. Through the course of infection a modest increase in IFNa and IFNc levels was observed in HK, low (~2 fold) but significant induction of *ifna* expression was observed at 14 dpi and a transient upregulation of *ifnc* was detected at 8 dpi. No significant induction of any of the interferons tested was observed in spleen, gill, or heart. In liver, IFNa and IFNc levels recovered by 7 dpi compared to 3 dpi but did not reach above baseline levels.

**Figure 5:**
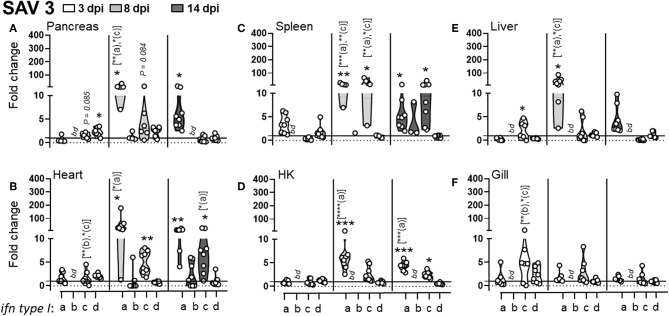
SAV3 infection elicit predominant type I IFNa and IFNc responses. Pre-smolt Atlantic salmon were intra peritoneal (i.p) injected with 100 μl SAV3 (1 × 10^5^ TCID_50_) or PBS and samples were collected at 3, 8, and 14 days post infection. Fold change of type I IFNa; IFNb: IFNc and IFNd in different tissues; **(A)** pancreas, **(B)** heart, **(C)** spleen, **(D)** head-kidney (HK), **(E)** liver, and **(F)** gill at different times post challenge [*n* ϵ (7;8)] relative to the control groups from the same time point (*n* ϵ 4) is shown. Each dot represents an individual fish and the line intersecting the y-axis at 1 represents the unstimulated control that the fold change of the treatments is in relation to. Asterisks above each violin plot indicates the strength of significance compared to PBS injected controls, and letters above each violin represent statistical differences among the different time points for each individual gene (*a* = 3 dpi; *b* = 8 dpi; and *c* = 14 dpi), *bd*, below detection, *p < 0.05, **p < 0.01, ***p = 0.0001, ****p < 0.0001. Primer sequences that are conserved for IFNa1 and IFNa2, for all IFNb and all IFNc subtypes were used (19).

Comparably, and consistent with previous studies (29), SAV3 infection induced a prominent type I IFNa response with the highest fold induction observed in heart (48-fold) and pancreas (15-fold) compared to control fish (Figure 5). Following SAV3 infection, type I IFNa expression in pancreas, spleen, and liver markedly peaked at 8 dpi, followed by a declined to moderate, albeit significantly, elevated levels by 14 dpi (Figures 5A,C,E). Comparably, IFNa levels in the heart and HK where maintained at high levels throughout the course of infection (Figures 5B,D). Moreover, SAV3 induced an increase in *ifnc* expression, which reached significant levels in the spleen and heart by 8 dpi. In contrast, *ifnb* transcript levels were below threshold levels in all uninfected organs and only reached detectable levels in the heart of infected fish at 14 dpi. In regard to *ifnd*, which is constitutively expressed in uninfected fish, a modest but significant increase in expression was observed in the pancreas and heart at 2 dpi, which returned to baseline by 8 dpi. It is noteworthy that in gill, that consistently showed no upregulation in L lineage gene expression, no induction of type I IFNs was detected (Figure 5F). Next, we compared the expression levels in individual fish (*n* = 8) to determine whether there was a correlation among induced L lineage genes and type I interferons following a SAV3 infection. In general, correlation between distinct L lineage genes (*lca, lga*, and *lha*) and IFN levels was observed only in pancreas and heart, the two main target organs for SAV3. Correlation between *lia* expression and *ifna* was apparent in spleen, pancreas and heart and for *ifnc* in the pancreas. Pearson correlation coefficients are given in sTable 2. Collectively, these data suggest that SAV3 infection results in an early (within 8 dpi) concerted and systemic transcriptional induction of type I IFNa and *lia*.

### MHC Class I L Lineage Induction in Primary Leucocytes Is Differentially Modulated in Response to Viral and Bacterial PAMPs

**Figure 6:**
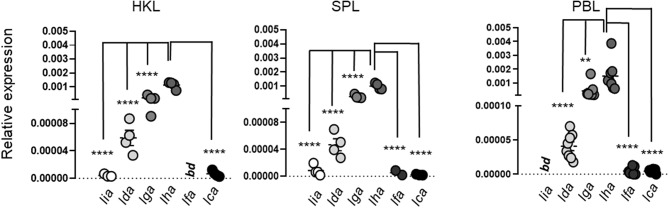
Relative expression of MHC class I L lineage genes in freshly isolated Atlantic salmon head kidney leucocytes (HKL), spleen leucocytes (SPL), and peripheral blood leucocytes (PBL). Gene expression was analyzed by qPCR [*n* ϵ (4;8)]. Data are presented as relative expression where the boxes indicate mean and SEM. Statistical significance between the different MHC class I L lineage expression levels is indicated by brackets and the asterisks indicates the strength of significance: **p < 0.01 and ****p < 0.001.

**Figure 7:**
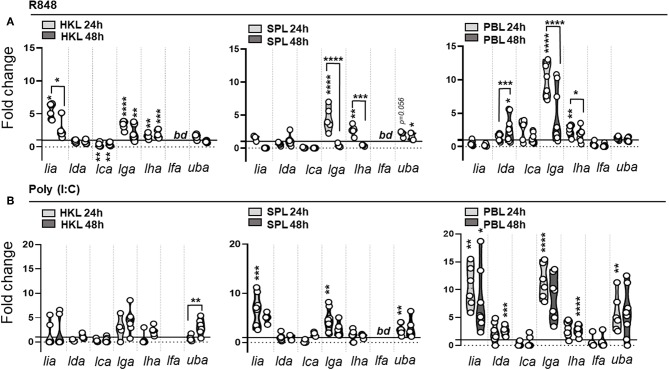
MHC class I L lineage mRNA induction in response to viral associated PAMPs in primary leucocytes. Expression of MHC class I L lineage (*lia, lda, lca, lga, lha*, and *lfa*) and classical MHC class I (*uba*) in HKL, SPL, and PBL stimulated with **(A)** 2μg R848 and **(B)** 10 μg Poly (I:C) for 24 h (light gray) and 48 h (dark gray Gene expression data at each time point were normalized against the reference gene EF1αB and fold changes were calculated using the unstimulated sample (media alone) at the same time point. The data represent values from [*n* ϵ (6;8)] individuals, with each dot representing cells isolated from an individual fish. The line intersecting the y-axis at 1 represents the unstimulated control that the fold change of the treatments is in relation to. Asterisks above each violin plot indicates the strength of significance: *p < 0.05, **p < 0.01, ***p < 0.001, and ****p < 0.0001. *bd*, below detection.

**Figure 8:**
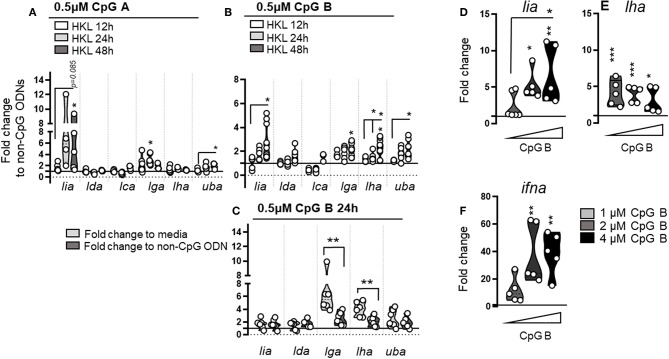
Stimulation with different classes of ODNs containing unmethylated CpG motifs induces lineage specific MHC class I L lineage expression. Expression of MHC class I L lineage (*lia, lda, lca, lga, lha*, and *lfa*) in HKL stimulated with 0.5 μM CpG A **(A)** or CpG B **(B)** ODNs for 12 h (white), 24 h (light gray), and 48 h (dark gray). Gene expression data at each time point were normalized against the reference gene EF1αB and fold changes were calculated using cells stimulated with non CpG containing ODN controls at the same time point. The data represents least five individuals [n ϵ (5;8)] with each dot representing cells isolated from an individual fish. **(C)** Fold change in gene expression calculated using cells stimulated with either media alone (light gray) or non-CpG containing ODNs (dark gray) at 24 hps. **(D–F)** Expression of *lia, lha*, and type I *ifna* in HKL stimulated with increasing concentrations of CpG B for 48 h. The line intersecting the y-axis at 1 represents the control that the fold change of the treatments is in relation to. Asterisks indicates the strength of significance between the indicated time points and asterisks above each violin indicate significant upregulation compared to control stimulated cells: *p < 0.05, **p < 0.001, ***p < 0.0001.

During the early phase of pathogen recognition the innate immune system detect a variety of conserved structural components, termed pathogen-associated molecular patterns (PAMPs), through germline encoded pattern recognition receptors (PRRs), such as Toll like receptors (TLR). To elucidate if different molecular components, common to many microbial pathogens, regulate the induction of distinct L lineage genes, we next determined how different viral (polyinosinic:polycytidylic acid [poly (I:C)] and R848) and bacterial (CpG ODNs) associated PAMPs influence L lineage gene transcription in primary leucocytes isolated from head kidney (HK), spleen and peripheral blood (PB). Figure 6 shows the basal expression of L lineage genes in unstimulated leucocytes isolated from head-kidney (HKL), spleen (SPL) and peripheral blood (PBL). *Lha, lga, lda*, and *lca* transcripts were detected in leucocytes isolated from all three cell populations. *Lfa* transcripts were consistently found in leucocytes isolated from peripheral blood and in a fraction of SPL but not in HKL, whereas *lia* transcripts were undetected in unstimulated PBL. Similar to whole tissue expression patterns *lha* and *lga* had the highest relative expression representative across all leucocyte populations while *lca* and *lia* expression was barely detectable. Prior to stimulation, a basal characterization of how L lineage *de novo* synthesis is altered *in vitro* was undertaken. With the exception of *lda*, 48 h incubation in 5% FBS supplemented cell culture media resulted in a 1-4-fold reduction in transcript levels compared to freshly isolated leucocytes (sFigure 5A). During this time, we also observed a reduction in cell viability, the percentage of live cells was on average reduced from > 95% in freshly isolated leucocytes to 72–82% after 48 h in culture (sFigure 5B). While the viability in stimulated cells was in the same range as unstimulated cells, the pattern of L lineage gene expression was dramatically altered by the addition of viral and bacterial mimics (Figures 7, 8). Stimulation with the imidazoquinoline R848, a viral single stranded (ss) RNA mimic that signals through TLR7 (36, 37), resulted in marked upregulation of *lga* and to a lesser extent *lha* transcript levels in all three leucocyte populations. In SPL and PBL, *lga* and *lha* transcript levels peaked at 24 h and then returned to basal levels by 48 h, while in HKL mRNA levels of both genes remained 2-3-fold elevated compared to unstimulated controls throughout the course of the experiment (Figure 7A). In contrast, no upregulation of *lia* was detected in SPL or PBL while in HKL *lia* expression was significantly elevated at 24 h followed by a decline in transcript levels by 48 h. Of note, a 2.7-fold increase in *lda* transcripts (*P* = 0.0122) was detected in PBL (but not in HKL or SPL) 48 h post stimulation. Similarly, extracellular addition of polyinosinic: polycytidylic acid (Poly [I:C]), a dsRNA mimic that binds TLR3 (38) and TLR22 (39) and induces IFNs via the viral RNA receptor MDA5 (40), resulted in elevated *de novo* synthesis of *lia, lga*, and *lha*. However, compared to R848 stimulation *lia* induction in response to poly I:C was more pronounced in SPL and PBL compared to HKL which might reflect the level of individual TLR expression in these tissues. Similarly, *lga* and *lha* were most prominently upregulated in PBL, with an > 11-fold increase for *lga* and a more modest >3-fold increase for *lha* compared to untreated cells 24 hps. No significant upregulation of *lga* nor *lha* was detected in poly [I:C] stimulated HKL. Of note, similar to R848 stimulation, poly [I:C] also induced a significant upregulation in *lda* transcript levels 48 hps strictly restricted to PBL.

Next we stimulated primary cells with different CpG oligonucleotides (ODNs), that mimic the structure of bacterial (and some viral) DNA. Similar to mammals, CpGs bind to TLR9 in Atl. salmon (41) and depending on the class (CpG A or CpG B), differ in their capacity to stimulate cells: CpG A induces higher amounts of IFNa, whereas CpG B stimulates more pronounced cell proliferation (42). Consistent with previous reports (42) *ifna, ifnb*, and *ifnc* expression was elevated in response to CpG ODNs with a significantly higher fold induction of all three interferons in the presence of CpG A compared with CpG B in HKL (data not shown). No induction of either type I interferons, L lineage genes, nor classical MHC class I was observed in CpG stimulated PBLs suggesting that the appropriate PRRs are missing in this cell population (data not shown). With regard to L lineage induction, upregulation of *lia* as well as classical MHC class I expression was evident in HKL following both CpG A and B stimulation. In general, the individual fold increase of *lia* was higher in CpG A treated cells, however, there was also a greater fish to fish variation precluding statistical significance. *Lga* gene expression was also modestly upregulated in response to both CpG A and CpG B. Of note, compared to media alone, *lga* transcript levels were 6-fold higher (compared to a 2-fold induction when normalized against non-CpG containing ODN controls; Figure 8C). This suggests that *lga* may be nonspecifically induced in response to extracellular gDNA, while *lia* respond more specifically to distinct CpG-ODN motifs. A different pattern emerged for *lha*, which was selectively upregulated following CpG B treatment, a small but significant transcriptional induction was apparent by 24 h post stimulation (hps) with low levels (0.5 μM) of CpG B that continued to rise by 48 hps. This induction pattern was further explored in a dose-response experiment. Changes in L lineage and *ifna* transcripts were measured 48 hps with increasing concentrations of CpG B. *Lia* transcript levels increased with higher concentrations of CpG B in parallel with a higher induction of *ifna* (Figures 8D,F). In contrast, *lha* transcript levels were consistently higher compared to controls but did not increase in response to higher amounts of CpG B. Increasing the concentration of CpG B did not change the induction potential of *lga* and no significant change was observed for either *lda* or *lca* in any of the treatment groups (data not shown). Collectively these data indicate that exposure to different viral and bacterial associated danger signals results in unique induction patterns of L lineage genes supporting the notion that these genes have distinct roles in the Atl. salmon immune defense.

### Lia and lga but Not lha Transcription Is Upregulated in Response to rIFNa

**Figure 9:**
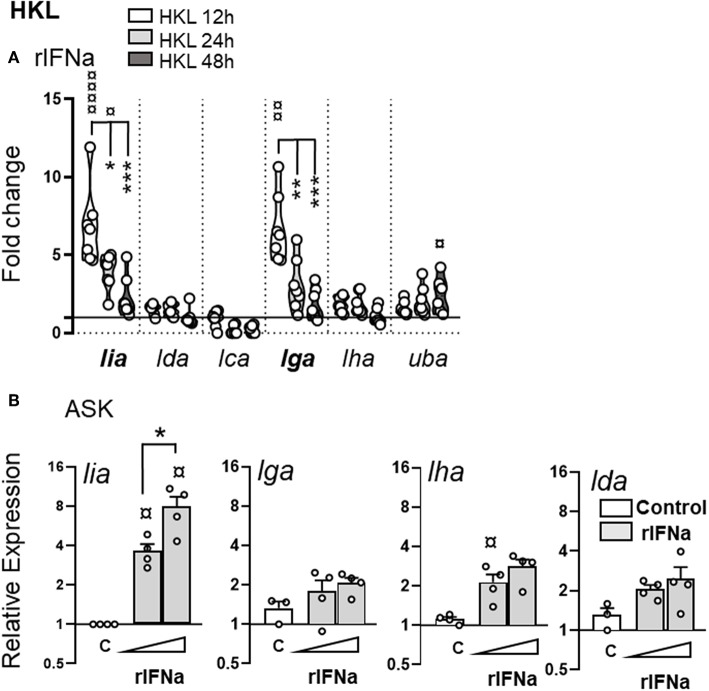
Stimulation with rIFNa upregulates lia and lga transcription. **(A)** Expression of MHC class I L lineage (*lia, lda, lca, lga, lha*, and *lfa*) in HKL 12 h (white), 24 h (light gray), and 48 h (dark gray) post stimulation with 500 U recombinant (r)IFNa. Gene expression data at each time point were normalized against the reference gene EF1αB and fold changes were calculated using control cells stimulated with media alone at the same time point. The data represents [n ϵ (6;8)] with each dot representing cells isolated from an individual fish. **(B)** Relative expression of *lia, lga, lha*, and *lda* in ASK cells stimulated with 200 U or 500 U rIFNa for 24 h. C, unstimulated cells. The line intersecting the y-axis at 1 represents the control that the fold change of the treatments is in relation to. Asterisks indicates the strength of significance between the indicated time points and ¤ indicate significant upregulation compared to control stimulated cells: *p < 0.05, **p < 0.001, ***p < 0.0001.

To determine if changes in L lineage transcriptional activity mainly reflect type I interferon driven induction versus the presence of a pathogen, the ability of IFNa to induce L lineage gene expression in the absence of any additional microbial stimuli was compared *in vitro*. HKL were treated with 500 U/ml of recombinant (r)IFNa and L lineage mRNA levels were measured by relative qPCR at different times (12, 24, and 48 hps) A marked upregulation of *lia* and *lga* was observed in IFNa treated cells, peaking at 12 h and then returning to baseline levels by 48 h (Figure 9A). A modest induction of classical MHC class I (*uba*) was also detected in response to rIFNa. As expected from the promoter region composition and the qPCR results from the challenge studies no induction of *lda* or *lca* was observed in response to rIFNs. However, more surprisingly, rIFNa also failed to induce detectable *de novo* synthesis of *lha*.

The induction of *lia* by IFNa was confirmed by analysis of Atlantic salmon ASK cells treated with 200 and 500 U/ml of rIFNa (Figure 9B). Unstimulated ASK cells express basal levels of *lga, lha*, and *lda* with no detectable *lia, lca*, or *lfa* transcripts. After 24 h, IFNa induced transcription of *lia*, with a significantly higher induction potential in ASK cells treated with 500 U/ml of IFNa compared to those treated with 200 U/ml (Figure 9B). Similar to HKL, no induction of *lha* or *lda* was observed in response to rIFNa. In contrast to HKL no induction of *lga* was observed in IFN treated ASK cells. Increasing the incubation time to 48 h did not result in any increase in L lineage expression (data not shown). Taken together, these experiments demonstrate that *lia* and *lga* transcription is markedly upregulated by type I IFNa, the signature cytokine of the Atl. salmon innate antiviral immune response.

## Discussion

Our identification of MHC class I L lineage genes as differentially regulated in response to type I IFNa, TLR ligands and pathogen challenge emphasizes the relevance of non-classical/MHC class I-like molecules in immune surveillance across jawed vertebrates (the main findings are summarized in Table 1). Among the six L lineage genes investigated, constitutive, and inducible expression patterns for each gene were unique, indicating differential functioning across the range of molecules, distinct from that of *Sasa-uba*- the sole classical MHC class I gene described in Atl. Salmon In particular the marked transcriptional induction of *lia* and *lga* in response to type I IFNa, along with the observation that SAV3, but not *P. salmonis*, results in systemic up-regulation of *lia*, while both viral and bacterial pathogens lead to upregulation of *lga* suggest that these MHC class I genes are critically involved in anti-microbial defenses. Further the preferential induction of *lha* in response to CpG B and R848 stimulation points to discrete roles of L lineage proteins in Atl. salmon immune responses, possibly as specialized antigen presenting molecules or alternatively as versatile indicators of cellular stress.

**Table 1:** Summary of the different proximal promoter response elements and transcriptional induction among Atl. salmon MHC class I L lineage genes.

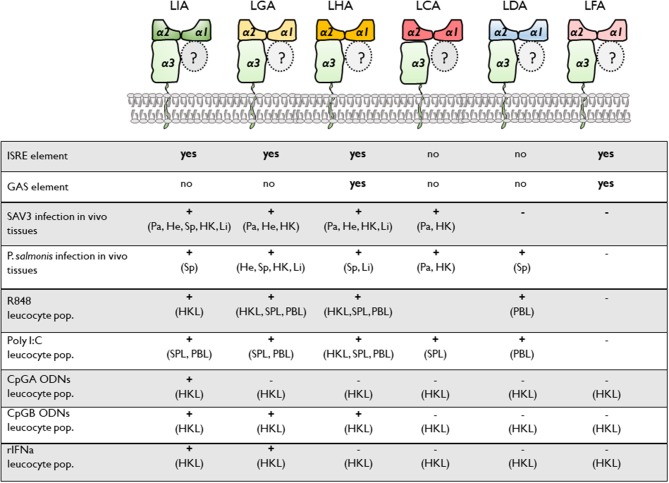

*The six different L lineage genes are schematically represented on top, whether or not these molecules associate with B2-microglbulin remain to be determined. For SAV3 and P. salmonis infections (+) indicates significantly higher expression in the indicated tissues compared to PBS injected fish, (–) indicates no change in expression. Tissues examined are as follows; Pa, pancreas; He, heart; Sp, spleen; HK, head kidney; Li, liver. For R848, Poly I:C, CPG and rIFNa (+) indicates significant upregulation in the indicated leucocyte cultures (HKL, head kidney leucocytes; SPL, spleen leucocytes; PBL, peripheral blood leucocytes)*.

Multiple lines of evidence imply a critical role of classical MHC class I in poikilothermic anti-viral responses, generally believed to function similarly to those of mammalian MHC class I (23–25, 43). Comparably, the possible contributions of MHC class I-like and non-classical MHC lineage genes in teleost immune responses remains largely unexplored. In this study, we focused on the divergent MHC class I L lineage genes and present a systematic assessment of their basal and inducible transcription patterns in response to various immune challenges. Among the six genes, *lia* was notable in that, while barely detectable in unstimulated cells, this gene was strongly upregulated in response to viral danger signals. Following SAV3 challenge *in vivo, de novo* synthesis of *lia* peaked prior to the onset of classical MHC class I transcription reaching a > 9-fold upregulation in spleen and liver eight days after SAV3 infection. At the same time *lia* was also upregulated in pancreas, heart and head kidney, albeit more modestly with a 3-4-fold increases compared to uninfected controls. In stark contrast i.p. infection with the intracellular bacterium *P. salmonis* resulted in minimal upregulation of *lia* gene expression. A likely explanation for this lack of induction drawn from the present data is that *P. salmonis* infection dampens the host type I interferon response. Another possibility is that *P. salmonis* in itself may have direct inhibiting effects on MHC expression (classical class I as well as L lineage genes). In support of this *P. salmonis* infection has been shown to lead to an altered transcriptional activity marked by down regulation of multiple genes critically involved in the adaptive immune response (44). Clearly these two explanations are not mutually exclusive and it is likely that the transcriptional induction of *lia*, as well as the other L lineage genes is multifaceted.

The transcriptional regulation of *lia* were further investigated *in vitro* by stimulating primary leucocytes with viral RNA mimics. Transcriptional induction of *lia* was apparent in HKL in response to stimulation with both R848 (an ssRNA mimic) and Poly I:C (a dsRNA viral mimic) suggesting that this gene might be involved in immune defense against viruses outside the ssRNA alphavirus family. However, in lieu of protein level molecular tools the full biological impact of this relatively modest fold-change in RNA expression is difficult to assess. Further, whether the observed upregulation of distinct MHC class I L lineage genes represents a universal transcriptional induction or a targeted induction in specific subsets of cells with high expression potential, remains to be determined. In comparison to classical MHC class I the fold change of specific L lineage genes is on par with what has been previously reported for Atl. Salmon. For example, 2-4-fold changes in expression of classical MHC class I was reported following challenge with both SAV (29, 30) and ISAV (45). More importantly, in humans a 3-4 fold increase in CD1c expression in tonsillar B cells resulted in a >13% increase in cell surface expression and significantly enhanced APC functions of the B cells (46).

Similar to *lia, lga* was also induced following R848 and Poly I:C stimulation as well as SAV3 challenge but with different kinetics and within distinct tissues and cell populations. For example, *lga* was upregulated in leucocytes isolated from HK, spleen and peripheral blood following R848 stimulation—with the highest induction observed in peripheral blood leucocytes (PBL). Comparably, *lia* transcription in response to R848 was only detected in HKL. Further, *lga* was also upregulated in response to both SAV3 and *P. salmonis* infections, suggesting that this gene might have a broader induction pattern compared to *lia*. Yet another pattern of induction was observed for *lha*, the most striking difference being that no induction of *lha* was found in HKL in response to rIFNa stimulation. This lack of induction may at first appear puzzling as *lha, lia* and *lga* all share the presence of interferon stimulating response elements in their 5′-untranslated region, distinct from *lda* and *lca*, which apparently lack these elements. Unlike *lia* and *lga*, however, the promoter region of *lha* also contains a conserved GAS element, which is the main promoter element for IFNγ induction, suggesting that *lha* may be preferentially induced by type II rather than type I interferons. A similar situation was recently shown to be true for Atl. salmon Mx genes, where a cluster of Mx genes responded more strongly to IFNγ than to type I IFNa (47). In mammals, both type I (IFN-α and IFN-β) and type II (IFN-γ) IFNs elevate MHC class I transcription and presentation. In a similar manner, induction of classical MHC class I and MHC class I pathway genes in salmonids has been demonstrated during anti-viral immune responses against different viruses, and there is cumulative evidence that type I IFNa signaling leads to induction of classical MHC class I pathway genes (23, 29, 30, 43, 45, 48). As Atl. salmon possess an extraordinary repertoire of type I IFN genes with several subtypes and a complexity that allows for a large variation in function, it is possible that *lha* transcription is selectively upregulated by another class of type I IFN. In support of this, there is convincing evidence that, in response to a viral infection, different Atl. salmon cell types produce different type I IFNs that may in turn induce genes in different cell types depending on their respective expression of IFN receptors (19).

Clearly, more studies are needed to elucidate the full impact of the various MHC class I L lineage genes in Atl. salmon immunity. However, it seems likely that that the potential roles for L lineage genes are varied and may include specialized roles for combating specific types of pathogens. Nevertheless, this raises the possibility that, assuming specific L lineage genes are involved in ligand interaction, cells that generally lack the ability to activate their cognate ligand gain this ability when they receive stimuli, leading to MHC class I surface expression. In parallel, peripheral antigen presenting cells in humans selectively express distinct CD1 genes in response to certain activating stimuli such as TLR agonists and certain cytokines. For example, TLR 2 recognition of polar lipids, shed from the cell wall of *Mycobacterium tuberculosis*, induced the expression of all three group I CD1 (CD1a, CD1b, and CD1c), but not CD1d, which is the only member of human group 2 CD1 proteins on monocytes (49). In contrast, the absolute CD1d surface expression can be modulated from intermediate toward high or low by different virus (50, 51) and bacteria (49, 52).

It is tempting to speculate that the nature of the microbial signal received by Atl. salmon L-lineage expressing cells dictates which specific genes are induced and may thus fine-tune the functional outcome of their subsequent crosstalk with effector cells. Viewed this way, one could imagine that the different MHC class I L lineage molecules interact with distinct ligands. Indeed, a possible role for *lia, lga* and *lha* molecules during infection is as antigen presenting molecules that bind to unconventional T cells such as NKT, iNKT, and innate T cells. For *lia* this is supported by the early induction pattern, indicating that LIA is expressed on the cell surface prior to the onset of the conventional T cell response. Another possibility is that MHC class I L lineage molecules are involved in regulation of NK-like cells through interaction with NK receptors. Teleost NK cell-like activity has been described in several fish species and several polymorphic multi-gene families, including the NITR (53–55) and LITR genes (56), have been proposed as candidate NK cell receptors. However, the interplay between different MHC class I lineage molecules and NK cell receptors in fish still remains elusive.

In conclusion our findings adhere to the paradigm that non-classical/MHC class I-like genes are critically involved in various aspects of the immune response. Indeed, they may represent a highly adaptable species-specific immune recognition system subject to strong co-evolutionary pressures among ligands on effector cells, MHC class I genes, and pathogenic ligands. In this study we used transcription as a proxy for functionality and while this is a logical first step, further studies will be needed to focus on protein translation, intracellular trafficking, and ultimately surface expression of the different MHC class I L lineage molecules. Further, while the present data indicates important roles for *lia, lga*, and *lha* during the early anti-viral immune response the potential involvement of these molecules during later stage infections, and in response to other microbes remains to be determined. To conclude, our studies provide compelling evidence that MHC class I L lineage genes are involved in various aspects of Atl. salmon immune responses.

## Data Availability Statement

The datasets generated for this study are available on request to the corresponding author.

## Ethics Statement

The animal study was reviewed and approved by Norwegian Animal Research Authority.

## Author Contributions

E-SE conceived and designed research. SS, AG-W, YW, and E-SE performed research and analyzed data. BR, IJ, and JJ contributed reagents (rIFNa, SAV3 RNA samples) and helped design experiments. SS and E-SE wrote the paper. All authors reviewed and approved the manuscript.

### Conflict of Interest

YW was employed by the company Vaxxinova GmbH. The remaining authors declare that the research was conducted in the absence of any commercial or financial relationships that could be construed as a potential conflict of interest.
