# Supplementary material for: Microbial Danger Signals Control Transcriptional Induction of Distinct MHC Class I L Lineage Genes in Atlantic Salmon
Source: Front Immunol. 2019 Oct 11;10:2425. doi: 10.3389/fimmu.2019.02425 (PMC6797598; doi:10.3389/fimmu.2019.02425)
Supplement: Supplementary file 1 [file Data_Sheet_1.docx]

***SUPPLEMENTAL INFORMATION***

******

***sFig1: Tissue specific MHC class I L lineage gene expression***. Tissues were collected from pre-smolt A. salmon, (n ϵ [5;6]) and are as follows: Thymus (Th), spleen (Sp), head kidney (Hk), kidney (Ki), gill (Gi), intestine (In), pyloric caecum (Pc), skin (Sk), dorsal fin (Df), adipose fin (Ad), heart (He), and liver (Li). Gene expression analysis of (A) *uba,* (B) *lia*, (C) *lda*, (D) *lca*, (E) *lga*, (F) *lha* and (G) *lfa* is shown. Results are normalized to a endogenous control (Ef1-αB) and presented as relative expression compared with the lowest observed expression of each gene (ΔΔCT). Each dot represent an individual fish and the overall distribution is represented by a violin plot. *P < 0,05, ** P<0,005, ***P<0,001 and **** P<0,0001 denotes statistical significance to the gene expression in the indicated tissues (one way ANOVA followed by post-hoc analysis using Tukey`s multiple comparisons test).

***sFig 2***

***A***

***LIA promoter***

GAAATGCCAGATGGCAGTCCGCCCCTGGTCAGCCTGGAGATGACAGGGAGTTACTATAGCGC

AGCTGTCTAGTTTTCATGGCTTGGGCTCTGGACACAAGGCCAG**aTTGTGAAAta**ATTTCCTT

***C/EBPβ***

AGGCAAGCTCTCAGAAATCATTTGACATGAAATGGCAATTGGATCAAATCAAACAGTGATAC

AAAACATAAGAATCCACACAGGAGAGTTTTTGTAAAATGAGAGCCTAATTTAACAAAAGAAT

AGCTTGGAACACACAAAATACTTTAATAGGCAGACCGAACATGTTTTTTCTCCTAAATTATC

ATTATTTAAATCCCTAATTCT**gtTTG***a***GAAAca**GTTTATTGCAGAT**ttG***t***CCAATGA***c***ag**C

***C/EBPα NF-Y box (CCAT binding factor)***

ATATACTAGGTGTTTGCCAA**GAAA*gt*GAAA**GTGTGTAAGGCTTAGT**GAAA*gt*GAAA**GTGTGC

***ISRE ISRE***

CAAATTATTTGAGAGCTGCCCTAGAAGCATATGAGCGTACAGAAAAGTATTGAACACATTCA

GAACC***ATG***

***B***

***LDA promoter***

TAAACCg**ACA**g**CTTT**ctAAACCTAGCAGACATATTGGAAACCTTATCTTACCGTGATTATAA

***C/EBPβ***

GAAATCCCGAGTCAAATCAACTTTTAAACAACTATCTGCCTGACATCGTTCCTGAGGAAGGC

GACTAGTAAAGTCAGTTGACAGCAGTTGTGCTGTGGTGTGCGGAACAAGCCATGGCGCGAGG

AGAAACAGCTCAGTGGTTTCGGTCTGCCTCTGTAGTTATATTTACATAAATAAACTCTCTGT

CCGGCAAGCGCACCTGCATATTTACATTTACAAAATTGTCAATATCGCgt**G***t***CCAATCG***c*tg

***NF-Y box (CCAT binding factor)***

CAGTCCACGAAATACCGGCGCACCTCGCCACGTCAGGAATGCAgg**AACCAATGGG**atGCTCG

***NF-Y box (CCAT binding factor)***

AGGGAGGGGCGTTCCTGACGCTGGAATGCCCACAGGCAGGAACGTAGTATATATTATTTTTA

***TATA***

ATAAGTGAAACAGGAAGTCTGTCAGAAACTTGAGACATGCTACACAAGGCTACACCTCAAAA

TC***ATG***

***C***

***LCA promoter***

TCAAAAGTAGTGCACTATATAGACAATAGGGTGCCATTTGGGATGCATACATTATCACTGGC

TTTAGTAATTCCAGTGACATACATTTTTTTCCTATAATCTCGCAAATCTCCGTTTTGGACGA

GACTGACTTTATGTCCAAAGTTATCCTATTTACACTTTGTTGTCAATTTTGACACTAGAATA

AACGTTTCTGACTCATATCGATGCCAC**ATTGG**CCATTTTCTAAACGAGAAGTTACTTTTAAG

***Y***  GG**ga**a**GTTGCTct**TTAATAACAGGAAGCATAGAAATAGCAGAAATGTCACAGATCTACTGCT

**RFX5**

TCTCAGACTTGCTACAATGAGAATGACAGATCTATAACTCATTTCTATGTGAATTTGGTATG

GTCTCCCAAAAAGCCACATAGCCAACTGCGTAGCATATTGTTTTTCATAGCTAAAAGAAGGG

GGAAGTCTGTCAGACACACTTTAGAGCtTAGaAACTTGCTACACGAGGAAACATTTTCTAAA

TC***ATG***

***D***

***LGA promoter***

GGTTGCAAGATCGAATCCCCGAGCTCACAAGGTAAAAATCTGTCGTTCTGCCCCTGAACAAG

GCAGTTAACCCACTGTTCCTAGGCCGTCATTGAAAATAAGAATTTGTTCTTAATTGACTTGC

CTAGTTAAATAAAATAAAAATAATAATAATGTTCTGCTGGCTTGCCCCACCACCAGAGAAAG

CAGTGAGCTAGGCTGAAACACCTGCATTTTGGAGCTGCCTTACTCAAGAAAACAAAAAAGAG

ACCATGTATGATGATTTATTAACTACATTTTATATAGATGTTTTTTTACATTGTTTGCAAAC

TGATATGTGACACGTATTAATGCCAAAAGAACATGCAAAACAGGCAAGCCCATTGCTCTGCC

CCACCTGCCCTGAATGACGGGTCGCC**ATTGG**CAGTTGGCTAAGT**GAAA*ga*GAAA**GTCTGACA

***Y ISRE***

GACTCACTTCTTGCTACGCAATCGGCTAAGTGGAAGGGAAAGTCTGACAGACTCACTTCTTG

CT***ATG***

***E***

***LHA promoter***

ACTCGTCATTATATCTGGTTGGCAAGCATACACATGGATTGTGTACGGAGTGTATGGTTGAG

AAACAGTGAAGCATGTGTTGATGTATTGTTCTAAGTACTGGTATGTGGAAGAGAGGAAAAGA

TTGACGTGTAGGGTTATTGAGGTAGGACAGGGATGGGGGGGTGGG**g***a***TGTGAAAgg**GATTTT

***C/EBP***

GGGGAATGGGGGAGGTTTTATAATAGTTAGTAGGGCTCTTTTTTATATTCTTAGTAGTACAG

GATTAGATAGGTTGGTTAAGTTTCACTTAATGTTTGTTTCTTTATTCATTTAGTCTGTAAAC

TGTGATTGACTACACACTCCAGTACAGTAGGTGGCGACATGCACCTCTAACATTTGTTTGCA

GACCGCCATAATA**TTC*ta*GAA**GAAAATAAGCAGAAGAAGAAGAAGAAGAAGAAGATAAGCTT

***GAS***

AAGT**GAAA*gg*GAAA**GTATCAGCTGCAGTGAGGCTACTGCAAAAGTAGTGAGCATTTCTAAAT

***ISRE***

C***ATG***

***F***

***LFA promoter***

TTCTTTAACTAGTGAAAAACACACTAAAACCAAATGAAAATAGACCACTCACAATAAATCAG

ACATATTTCCACGACAATATTCTGAATTATGTCTGTATTCTGCTTGTATATTGTCTATTGCT

GTCAGCACCTTCTCAGTAAGGTAAATTCTGGGTATGTGTAAATGTACTTGACGAATATATGT

GATACTGA**TTC*ta*GAA**CCTTACATTACTGAACACAGAACATCCTCAGTGTTTTAAACTAGAA

***GAS***

CCTTAAATTACTGAACACAGAACATCCTCAGTGTTTTAAAGCTAAAGCAGTCTCTTCCTCAT

TCCATTCTCAGTGCATATCAGTTTGAATTCCCACCACCACTTGTTTGACCTGTACTTGTTTG

ACTTGTTCAACAACTTTAAAGTGCCTTACCTTTTTTTGCTAAGC**GAAA*g*GAAA**AGTCTGTCA

***ISRE***

GACTCACTTGAGAGCTAGA**AACCTG**CTACGCGAGGCT**ATTGG**AAAAAAGTGAACACTCAAAG

***S/W Y***

TC***ATG***

***sFig 2. Sequence analysisis of the promoter regions of MHC class I-like L lineage genes.*** The proximal promoter region of (A) lia, (B) lda, (C) lca, (D) lga, (E) lha and (F) lfa is shown. Putative transcription factor binding sites are indicated. Enhance elemnts including IRSE and GAS are shown in bold.

***sFig3. SAV3 transcripts in different tissues of Atlantic salmon following i.p SAV3 challenge*.** Pre-smolt Atlantic salmon were intra peritoneal (i.p) injected with 100 µl SAV3 (1 x 10^5^ TCID_50_) ­­­­ and samples were collected at 3, 8 and 14 days post infection. Viral loads and dissemination were determined using primers specific to the SAV3 non-structural protein 1 (nsP1). (A) Relative expression was determined using the 2^−ΔCq^ method after normalized against the host gene EF1-αB, (n ϵ 8) per time point. Each dot represents an individual fish and asterisks above each violin plot indicates the strength of significance using Mann Whitney U test *p < 0.05, **p<0.01, *** p<0.001 between the different time points. No virial transcripts were detected in any of the PBS injected control fish or at the 3 dpi time point. (B) Mean and standard error of Ct values for nsP1 expression. The number of fish with detectable nsP1 gene expression are indicated at the different timepoints. Samples were analyzed in duplicates with internal controls to control for inter-plate variations

***sFig4. 16S transcripts in different tissues of Atlantic salmon following i.p P. salmonis challenge*.** Pre-smolt Atlantic salmon were intra peritoneal (i.p) injected with 3 x 10^6^ CFU P. *salmonis­­­­* or PBS and samples were collected at 2, 7 and 14 days post infection. Bacteria loads were determined using primers specific to the P. *salmonis* 16S transcript. (A) Relative expression was determined using the 2^−ΔCq^ method after normalized against the host gene EF1-αB, n ϵ 6 fish per time point. Each dot represents an individual fish and asterisks above each violin plot indicates the strength of significance using Mann Whitney U test *p < 0.05 between the different time points. No bacterial transcripts were detected in the PBS injected control fish. (B) Mean and standard error of Ct values for 16S expression. The number of fish with detectable 16Sgene expression are indicated. Samples were analyzed in duplicates with internal controls to control for inter-plate variations.

***sFig 5***. ***Relative expression of L lineage genes in Atlantic salmon primary leucocyte 48 hours post isolation.*** (A) Log 2 fold change in L lineage gene expression in head kidney leucocytes (HKL), spleen leucocytes (SPL) and peripheral blood leucocytes (PBL) following 48h incubation in L-15 media supplemented with 4 % FBS compared to freshly isolated leucocytes. Ud = the number of fish with undetermined expression of the indicated gene. The dotted line intersecting the Y axis at zero represents the value of expression in freshly isolated leucocytes. (B) Viability of cells immediately after isolation (0) and after 48h in culture.
