# Supplementary material for: Microbial Danger Signals Control Transcriptional Induction of Distinct MHC Class I L Lineage Genes in Atlantic Salmon
Source: Front Immunol. 2019 Oct 11;10:2425. doi: 10.3389/fimmu.2019.02425 (PMC6797598; doi:10.3389/fimmu.2019.02425)
Supplement: Supplementary file 2 [file Data_Sheet_2.docx]

**sText1: Sequence read analysis of MHC class I L lineage genes in (A) heart from pre-smolt Atl. salmon challenged with SAV3 and (B) head-kidney, gill and liver from adult Atl. salmon challenged with infectious salmon anemia virus (ISAV).**

**A.** **Comparison of fold change in expression of MHC class I L lineage genes in heart during SAV3 infection.** Fold changes were estimated by transcriptome analysis of 35 sequence read analysis (SRA) sets derived as described in Bioproject PRJNA543940. In this study, sequence data collected from heart of pre-smolt Atl. Salmon with high genomic breeding values four and ten weeks post SAV3 infection were analyzed. RPKM (Reads per kilobase per million) were calculated based on hits that mapped with high stringency (i.e. greater than 98% identity) using the BLASTn interface. Fold change ± S.E in expression of L lineage genes (*lia, lga, lha, lda, lca* and *lfa*) at various times post challenge (n ϵ 10) relative to the uninfected control group (n ϵ 10) is shown. Statistical analysis was performed using one way ANOVA followed by Tukey`s multiple comparisons test, *p* values are indicated in the table, no significant change in expression was detected for any of the L lineage genes analyzed. wpi = week post infection.

sTable3. Comparison of fold change in expression of L lineage genes in heart following SAV3 infection.

| Gene name/wpi | 4 wpi | *p*-Value  W0 vs. W4 | 10 wpi | *p*-Value  W0 vs. W10 | *p*-Value  W0 vs. W10 |
| --- | --- | --- | --- | --- | --- |
| *lia* | 1,31 ±0.34 | *0.7802* | 1,19 ±0.18 | *0.9260* | *0.9516* |
| *lga* | 1,65 ±0.47 | *0.2905* | 0,91 ±0.28 | *0.9868* | *0.3649* |
| *lha* | 1,40 ±0.26 | *0.4309* | 0,96 ±0.16 | *0.9940* | *0.3744* |
| *lda* | 1,18 ±0.25 | *0.7670* | 1,10 ±0.37 | *0.8694* | *0.9799* |
| *lca* | 1,91 ±0.54 | *0.4019* | 1,54 ±0.51 | *0.7395* | *0.8393* |
| *lfa* | 1,68 ±0.43 | *0.2541* | 0,85 ±0.22 | *0.9760* | *0.1786* |

**B. Comparison of fold change in expression of Atl. Salmon L lineage genes in response to infectious salmon anemia virus (ISAV).** Fold changes were estimated by transcriptome analysis of 12 SRA sets derived as described in Bioproject PRJNA472087, originally reported by Valenzuela- Miranda et al (21). T1 = 3 days post confirmed ISAV outbreak (i.e three days after three consecutive days of dead trojans); T2 = 7 days post confirmed ISAV outbreak; and T3 =14 days post confirmed ISAV outbreak. RPKM were calculated based on hits that mapped with high stringency (i.e. greater than 98% identity) using the BLASTn interface and fold change were calculated compared to uninfected fish. Tissues with fold changes above two are shaded yellow. * For *lia* no reads with >98% identity were detected in the liver of unchallenged fish, RPKM at T1, T2 and T3 were as follows: 0,93, 2,52 and 0,81.

sTable 4. Comparison of fold change in expression of L lineage genes in response to ISAV.

| **Tissue/Gene** | **Head Kidney T1** | **Head Kidney T2** | **Head Kidney T3** |
| --- | --- | --- | --- |
| *lia* | 5,33 | 4,47 | 4,25 |
| *lga* | 1,58 | 0,70 | 1,14 |
| *lha* | 0,98 | 0,69 | 0,88 |
| *lda* | 1,28 | 1,02 | 1,11 |
| *lca* | 0,86 | 0,52 | 0,57 |
| *lfa* | 0,96 | 0,54 | 0,45 |
|  | **Gill T1** | **Gill T2** | **Gill T3** |
| *lia* | 1,82 | 1,69 | 1,34 |
| *lga* | 1,33 | 0,96 | 1,23 |
| *lha* | 1,07 | 0,90 | 1,02 |
| *lda* | 0,82 | 0,58 | 0,84 |
| *lca* | 1,09 | 0,72 | 1,26 |
| *lfa* | 1,00 | 0,91 | 1,05 |
|  | **Liver T1** | **Liver T2** | **Liver T3** |
| *lia* | * | * | * |
| *lga* | 3,4 | 4,6 | 2,6 |
| *lha* | 1,1 | 2,9 | 1,2 |
| *lda* | 1,4 | 0,8 | 1,2 |
| *lca* | 1,8 | 2,8 | 1,4 |
| *lfa* | 1,8 | 5,6 | 1,2 |

|  |  |  |
| --- | --- | --- |
|  |  |  |
