# Supplementary material for: Microbial Danger Signals Control Transcriptional Induction of Distinct MHC Class I L Lineage Genes in Atlantic Salmon
Source: Front Immunol. 2019 Oct 11;10:2425. doi: 10.3389/fimmu.2019.02425 (PMC6797598; doi:10.3389/fimmu.2019.02425)
Supplement: Supplementary file 3 [file Data_Sheet_3.docx]

**sTABLE1.** List of primers used in this study

| Name | Sequence | Accession Number, ref |
| --- | --- | --- |
|  | | |
| Ef1αB F  Ef1αB R | CCCCTCCAGGACGTTTACAAA  CACACGGCCCACAGGTACA |  |
| *Sasa-uba* F  *Sasa-uba* R | GACAGTGACACAGCTCAGAAT  CATCAGAGTGCTCTTCCCATAG | AAN75113; this study |
| *Sasa-lca* F  *Sasa-lca* R | GAAGCTAAGAACGGAGATTTTG   AGATTCAAGTGGTGCTTTAGGT | XP_013983104.1; this study |
| *Sasa-lda* F  *Sasa-lda* R | AGTTCTCTCTCTTCAGGCATTTC  TTGGATCAGGCAAGCAACT | XP_013989407.1; this study |
| *Sasa-lfa* F  *Sasa-lfa* R | TGGCATTTTTGTAGATCATGCG  TCATATGCTTGCCTTATCCCATA | XP_014031975.1; this study |
| *Sasa-lga* F  *Sasa-lga* R | CACAAAAACCAAGGACGATGAA   CGGTGCTTTAGTTCAAATGATCTG | AXP_014031973.1; this study |
| *Sasa-lha* F  *Sasa-lha* R | CAGCCCTGATCATGTCAAAGA  AAGATATTCTCTCCTCATCGCA | XP_014032010.1; this study |
| *Sasa-lia* F  *Sasa-lia* R | TCAGACCTGAGGTATCGCTTTA  AGCATCTGTGGACAGAATAAG | XP_014019802.1; this study |
| *ifna* F  *ifna* R | CCTTTCCCTGCTGGACCA  TGTCTGTAAAGGGATGTTGGGAAAA | DQ354152.1; [18] |
| *ifnb* F  *ifnb* R | TGCATTGGAGGCTATGCGATAT  TTCCCAAACACCACCTACGACA | EU768890; [18] |
| *ifnc* F  *ifnc* R | ATGTATGATGGGCAGTGTGG  CCAGGCGCAGTAACTGAAAT | EU768890; [18] |
| *ifnd F*  *ifnd R* | AACCCTTGACAAGATCGCTGAACTGTT  AGGCCGTCGGACTGACTGTTGAG | JX524151;[18] |
| *nsP1 F*  *nsP1 R* | AGTTCCAGACTGCGTTTCC  GGTAGCCAAGTGGGAGAAAG |  |
| *16S F*  *16S R* | AGGGAGACTGCCGGTGATA  ACTACGAGGCGCTTTCTCA |  |

**sTABLE 2.**

Significant correlation between MHC class I-like genes and SAV3, IFNa and IFNc at 7 dpi was determined using the Pearson Correlation coefficiency calculated from the relative expression of each gene normalized to EF1-αB *p < 0.05, **p<0.01, ***p=0.0001, ****<0.0001, ns = no significance.

| Genes | Spleen | HK | Pancreas | Heart | Liver |
| --- | --- | --- | --- | --- | --- |
| **(SAV3)**  lia  lda  lca  lga  lha  ifna  ifnc  ifng | 0.66/0.08*  ns  ns  ns  ns  0.82/0.045*  0.82/0.044*  0.94/0.006*** | 0.78/0.0039**  ns  ns  ns  ns  ns  ns  0.83/0.0016** | 0.89/0.0003***  0.68/0.02*  0.79/0.003**  ns  0.88/0.0005***  0.66/0.0145*  0.94/<0.0001****  0.86/0.009*** | 0.54/0.04*  ns  ns  0.63/0.019*  057/0.03*  ns (0.48/0.054)  ns  ns | 0.84/0.0017**  ns  ns  0.79/0.0029**  0.78/0.003**  ns  ns  ns |
| **(ifna)**  lia  lda  lca  lga  lha | 0.76/0.023*  ns  ns  ns  ns | ns  ns  ns  ns  ns | 0.77/0.00044**  ns  0.55/0.034*  ns  0.71/0.0092** | 0.88/0.0006***  ns  0.83/0.02*  0.69/0.01**  0.75/0.0054** | ns  ns  ns  ns  ns |
| **(ifnc)**  lia  lda  lca  lga  lha | ns  ns  ns  ns  ns | ns  ns  ns  ns  ns | 0.84/0.0013**  ns  0.87/0.0008***  ns  0.82/0.0016** | ns  ns  ns  ns  ns | ns  ns  ns  ns  ns |
